# The Recrudescence of Mrs. Eddy

**Published:** 1906-12

**Authors:** 


### THE RECRUDESCENCE OF MRS. EDDY.

AN event of interest to Christian Scientists and others was the appearance of the ancient dame who heads the cult in denial of allegations of the *New York World* that she was a senile wreck, cancerous and that some other person was substituted in her daily drives. Eleven reporters met at her residence, a portiere was swept aside, and the superannuated author of a volume which has guided thousands into paths of pure reason and crystal science appeared in all the girlish loveliness of her eighty-six years, standing at the foot of the stairs. To questions, she replied that she was in perfect health, had no physician but God and that she drove out each day. The reporters averred that she looked her age, which means that Christian Science denies material existence, but remorseless time goes on with his work of disintegration and decay and hide as she may time has found her out and put to flight her fine-spun sophistries.

And so will he deal with all that is false and unsubstantial as he has dealt with greater things in the past. Nothing remains of the elaborate polytheism of the Egyptians or of the

Olympus of the Romans but broken columns, noseless gods and jackal-infested temples. Will Christian Science leave even these poor relics? What has become of the theosophists, to take a modern instance, who twenty years ago gained many converts? The once flourishing cult is represented by a scant handful of the faithful collected at Point Loma, California, literally almost pushed into the sea, and the wraiths of William Q. Judge and Madame Blavatsky must find their sphere of spirit migration rather narrow.

Probably in a few more years, the doddering old widow of Concord will be called to her reward, whatever that is, and what will then be the fate of Christian Science can only be conjectured by the aid of precedent.

---

E. G. SWIFT announces that January 1, 1907, the *Medical Age* and *Medicine* will be consolidated with the *Therapeutic Gazette*, the oldest, strongest and most widely circulated of his three medical journals. Drs. Hobart A. Hare and Edward Martin will edit the consolidated journal and undoubtedly under their editorship this journal will be one of the most excellent monthly journals in the United States as regards practical therapeutics. The title of the new journal will be the *Therapeutic Gazette*, incorporating *Medicine* and *The Medical Age.* We wish them much success and believe this "concentration" will be sure to attain it.

---

DR. JOHN J. HILL died after an illness of three weeks at his home in Washington, Ga., November 12, aged fifty-four. Dr. Hill was a graduate of the College of Physicians and Surgeons of New York, and during the twenty years of his professional life he worked his way to the forefront of his profession.

